# Poly[[diaqua­bis[μ-(2,4-dichloro­phen­oxy)acetato]calcium(II)] monohydrate]

**DOI:** 10.1107/S1600536808009379

**Published:** 2008-04-10

**Authors:** Wen-Dong Song, Xiang-Hu Huang, Jian-Bin Yan, De-Yun Ma

**Affiliations:** a College of Science, Guang Dong Ocean University, Zhanjiang 524088, People’s Republic of China; b College of Fisheries, Guang Dong Ocean University, Zhan Jiang 524088, People’s Republic of China; c College of Chemistry, South China University of Technology, Guangzhou 510640, People’s Republic of China

## Abstract

In the title coordination polymer, {[Ca(C_8_H_5_Cl_2_O_3_)_2_(H_2_O)_2_]·H_2_O}_*n*_, the Ca^II^ atom is eight-coordinated by six O atoms from four different (2,4-dichloro­phen­oxy)acetate ligands and two water mol­ecules, and displays a distorted square-anti­prismatic coordination geometry. The compound forms an infinite zigzag chain through connection of the metal centers by (2,4-dichlorphen­oxy)acetate ligands and hydrogen bonding of coordinated and inter­stitial water mol­ecules. These chains are further hydrogen bonded with neighboring chains, forming a supra­molecular network.

## Related literature

For related literature, see: Song *et al.* (2006 ▶); Hao *et al.* (2006 ▶).
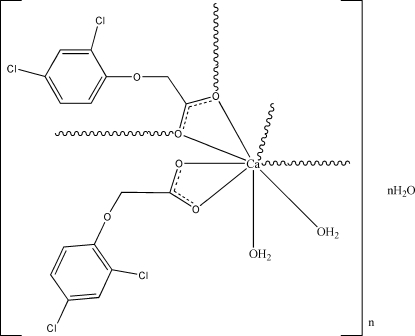


## Experimental

### 

#### Crystal data

[Ca(C_8_H_5_Cl_2_O_3_)_2_(H_2_O)_2_]·H_2_O
*M* *_r_* = 534.17
Monoclinic, 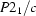

*a* = 17.8354 (7) Å
*b* = 6.8077 (3) Å
*c* = 18.5276 (8) Å
β = 101.297 (3)°
*V* = 2206.00 (16) Å^3^
*Z* = 4
Mo *K*α radiation
μ = 0.81 mm^−1^
*T* = 296 (2) K
0.30 × 0.26 × 0.23 mm

#### Data collection

Bruker APEXII area-detector diffractometer
Absorption correction: multi-scan (*SADABS*; Sheldrick, 1996 ▶) *T*_min_ = 0.790, *T*_max_ = 0.840
15522 measured reflections
5049 independent reflections
2962 reflections with *I* > 2σ(*I*)
*R*_int_ = 0.047

#### Refinement

*R*[*F* ^2^ > 2σ(*F* ^2^)] = 0.050
*wR*(*F* ^2^) = 0.158
*S* = 1.00
5049 reflections
289 parameters
9 restraints
H atoms treated by a mixture of independent and constrained refinement
Δρ_max_ = 0.55 e Å^−3^
Δρ_min_ = −0.60 e Å^−3^

### 

Data collection: *APEX2* (Bruker, 2004 ▶); cell refinement: *SAINT* (Bruker, 2004 ▶); data reduction: *SAINT*; program(s) used to solve structure: *SHELXS97* (Sheldrick, 2008 ▶); program(s) used to refine structure: *SHELXL97* (Sheldrick, 2008 ▶); molecular graphics: *XP* in *SHELXTL* (Sheldrick, 2008 ▶); software used to prepare material for publication: *SHELXTL*.

## Supplementary Material

Crystal structure: contains datablocks I, global. DOI: 10.1107/S1600536808009379/zl2102sup1.cif

Structure factors: contains datablocks I. DOI: 10.1107/S1600536808009379/zl2102Isup2.hkl

Additional supplementary materials:  crystallographic information; 3D view; checkCIF report

## Figures and Tables

**Table 1:** Hydrogen-bond geometry (Å, °)

*D*—H⋯*A*	*D*—H	H⋯*A*	*D*⋯*A*	*D*—H⋯*A*
O3*W*—H6*W*⋯O2^i^	0.848 (10)	2.176 (14)	3.013 (4)	169 (3)
O2*W*—H4*W*⋯O2^ii^	0.823 (10)	2.079 (16)	2.866 (3)	160 (4)
O2*W*—H3*W*⋯O1^iii^	0.822 (10)	2.205 (18)	2.986 (4)	159 (4)
O1*W*—H1*W*⋯O3*W*^iv^	0.823 (10)	1.927 (11)	2.745 (4)	173 (4)
O3*W*—H5*W*⋯O1	0.850 (10)	1.986 (12)	2.830 (4)	172 (5)
O1*W*—H2*W*⋯Cl4	0.818 (10)	2.88 (2)	3.530 (3)	138 (3)
O1*W*—H2*W*⋯O6	0.818 (10)	2.20 (2)	2.938 (3)	150 (4)

Symmetry codes: (i) 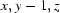; (ii) 
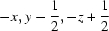
; (iii) 
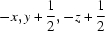
; (iv) 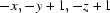.

**Fig. 1.**
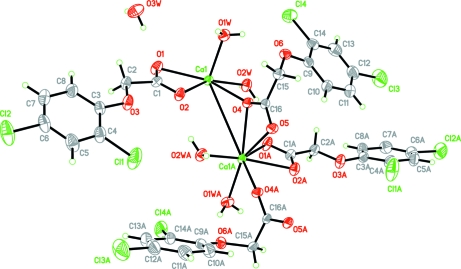
The structure of (I), showing the atomic numbering scheme. Non-H atoms are shown as 30% probability displacement ellipsoids.

**Fig. 2.**
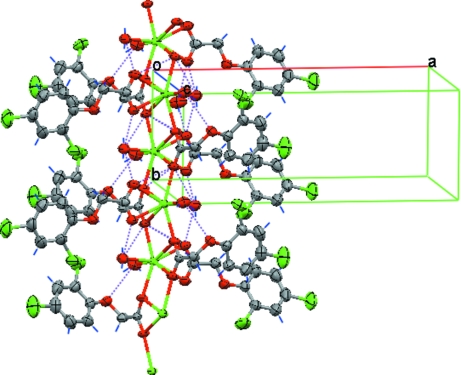
A packing view of (I).

## supplementary crystallographic information

### Comment

In the structural investigation of 2,4-dichlorophenoxyacetate complexes, it has been found that the (2,4-dichlorphenoxy)acetate functions as a multidentate ligand [Song *et al.* (2006); Hao *et al.* (2006)], with versatile binding and coordination modes. In this paper, we report the crystal structure of the title compound, (I), a new Ca complex obtained by the reaction of (2,4-dichlorphenoxy)acetate and calcium chloride in an alkaline aqueous solution.

As illustrated in Figure 1, the Ca^II^ atom exists in a distorted square-antiprismatic environment, defined by six O atoms from four different 2,4-dichlorophenoxyacetate ligands and two water molecules. The 2,4-dichlorophenoxyacetate ligands link the calcium ions to form infinite zigzag like chains, which are further stabilized by hydrogen bonding of the coordinated and interstitial water molecules O2W and O3W to carboxylate oxygen atoms (Table 1, Fig. 2). O1W, *via* a hydrogen bond to the ether oxygen atom O6, also stabilizes the chains, but also forms another intermolecular hydrogen bond to a water molecule O3W that is part of a neighboring chain, thus forming a supramolecular network of H-bonded chains.

### Experimental

A mixture of calcium chloride (1 mmol), 2,4-dichlorophenoxyacetate (1 mmol), NaOH (1.5 mmol) and H_2_O (12 ml) was placed in a 23 ml Teflon reactor, which was heated to 433 K for three days and then cooled to room temperature at a rate of 10 K h^-1^. The crystals obtained were washed with water and dryed in air.

### Refinement

Carbon-bound H atoms were placed in calculated positions and were treated as riding on the parent C atoms with C—H = 0.93–0.97 Å, and with *U*_iso_(H) = 1.2 *U*_eq_(C). Water H atoms were tentatively located in difference Fourier maps and were refined with distance restraints of O–H = 0.84 Å and H···H = 1.39 Å, each within a standard deviation of 0.01 Å, and with *U*_iso_(H) = 1.5 *U*_eq_(O)

### Crystal data

**Table:**

[Ca(C_8_H_5_Cl_2_O_3_)_2_(H_2_O)_2_]·H_2_O	*F*_000_ = 1088
*M**_r_* = 534.17	*D*_x_ = 1.608 Mg m^−^^3^
Monoclinic, *P*2_1_/*c*	Mo *K*α radiation λ = 0.71073 Å
Hall symbol: -P 2ybc	Cell parameters from 5837 reflections
*a* = 17.8354 (7) Å	θ = 2.8–27.9º
*b* = 6.8077 (3) Å	µ = 0.81 mm^−^^1^
*c* = 18.5276 (8) Å	*T* = 296 (2) K
β = 101.297 (3)º	Block, colorless
*V* = 2206.00 (16) Å^3^	0.30 × 0.26 × 0.23 mm
*Z* = 4	

### Data collection

**Table:**

Bruker APEXII area-detector diffractometer	5049 independent reflections
Radiation source: fine-focus sealed tube	2962 reflections with *I* > 2σ(*I*)
Monochromator: graphite	*R*_int_ = 0.047
*T* = 296(2) K	θ_max_ = 27.5º
φ and ω scans	θ_min_ = 1.2º
Absorption correction: multi-scan(SADABS; Sheldrick, 1996)	*h* = −22→23
*T*_min_ = 0.790, *T*_max_ = 0.840	*k* = −8→8
15522 measured reflections	*l* = −23→24

### Refinement

**Table:**

Refinement on *F*^2^	Secondary atom site location: difference Fourier map
Least-squares matrix: full	Hydrogen site location: inferred from neighbouring sites
*R*[*F*^2^ > 2σ(*F*^2^)] = 0.050	H atoms treated by a mixture of independent and constrained refinement
*wR*(*F*^2^) = 0.158	*w* = 1/[σ^2^(*F*_o_^2^) + (0.0794*P*)^2^] where *P* = (*F*_o_^2^ + 2*F*_c_^2^)/3
*S* = 1.00	(Δ/σ)_max_ < 0.001
5049 reflections	Δρ_max_ = 0.55 e Å^−^^3^
289 parameters	Δρ_min_ = −0.60 e Å^−^^3^
9 restraints	Extinction correction: none
Primary atom site location: structure-invariant direct methods	

### Special details

**Table:**

Geometry. All e.s.d.'s (except the e.s.d. in the dihedral angle between two l.s. planes) are estimated using the full covariance matrix. The cell e.s.d.'s are taken into account individually in the estimation of e.s.d.'s in distances, angles and torsion angles; correlations between e.s.d.'s in cell parameters are only used when they are defined by crystal symmetry. An approximate (isotropic) treatment of cell e.s.d.'s is used for estimating e.s.d.'s involving l.s. planes.
Refinement. Refinement of *F*^2^ against ALL reflections. The weighted *R*-factor *wR* and goodness of fit *S* are based on *F*^2^, conventional *R*-factors *R* are based on *F*, with *F* set to zero for negative *F*^2^. The threshold expression of *F*^2^ > σ(*F*^2^) is used only for calculating *R*-factors(gt) *etc*. and is not relevant to the choice of reflections for refinement. *R*-factors based on *F*^2^ are statistically about twice as large as those based on *F*, and *R*- factors based on ALL data will be even larger.

### Fractional atomic coordinates and isotropic or equivalent isotropic displacement parameters (Å^2^)

**Table:**

	*x*	*y*	*z*	*U*_iso_*/*U*_eq_	
C1	0.10806 (18)	0.6762 (5)	0.41264 (16)	0.0380 (7)	
C2	0.18125 (18)	0.6618 (5)	0.46951 (19)	0.0450 (8)	
H2A	0.1707	0.6023	0.5140	0.054*	
H2B	0.2175	0.5790	0.4510	0.054*	
C3	0.28244 (17)	0.8609 (6)	0.53280 (18)	0.0448 (9)	
C4	0.3216 (2)	1.0370 (6)	0.5327 (2)	0.0570 (10)	
C5	0.3898 (2)	1.0689 (7)	0.5798 (3)	0.0700 (13)	
H5	0.4156	1.1874	0.5791	0.084*	
C6	0.4196 (2)	0.9231 (7)	0.6281 (2)	0.0621 (11)	
C7	0.3831 (2)	0.7454 (7)	0.6279 (2)	0.0622 (11)	
H7	0.4046	0.6463	0.6600	0.075*	
C8	0.31519 (19)	0.7145 (6)	0.5804 (2)	0.0555 (10)	
H8	0.2908	0.5937	0.5801	0.067*	
C9	−0.24793 (17)	1.0577 (5)	0.26633 (18)	0.0408 (8)	
C10	−0.2516 (2)	1.2253 (6)	0.2240 (2)	0.0529 (10)	
H10	−0.2156	1.3243	0.2373	0.063*	
C11	−0.3083 (2)	1.2475 (7)	0.1617 (2)	0.0632 (11)	
H11	−0.3112	1.3622	0.1340	0.076*	
C12	−0.3600 (2)	1.1003 (8)	0.1412 (2)	0.0655 (12)	
C13	−0.3568 (2)	0.9291 (7)	0.1815 (2)	0.0638 (12)	
H13	−0.3919	0.8289	0.1668	0.077*	
C14	−0.30089 (18)	0.9088 (6)	0.2439 (2)	0.0481 (9)	
C15	−0.13933 (18)	1.1724 (5)	0.35198 (17)	0.0400 (8)	
H15A	−0.1135	1.1485	0.4023	0.048*	
H15B	−0.1637	1.3001	0.3502	0.048*	
C16	−0.08085 (15)	1.1739 (5)	0.30233 (15)	0.0290 (6)	
Ca1	−0.02416 (3)	0.67481 (9)	0.29818 (3)	0.03103 (18)	
Cl1	0.28379 (8)	1.21602 (17)	0.46954 (10)	0.1105 (6)	
Cl2	0.50445 (6)	0.9670 (2)	0.69082 (8)	0.1050 (5)	
Cl3	−0.43126 (7)	1.1255 (3)	0.06300 (7)	0.1062 (5)	
Cl4	−0.29530 (6)	0.69273 (16)	0.29402 (7)	0.0722 (3)	
O1	0.07745 (13)	0.5164 (3)	0.39084 (12)	0.0501 (6)	
O2	0.08134 (12)	0.8394 (3)	0.38986 (12)	0.0456 (6)	
O3	0.21318 (13)	0.8512 (4)	0.48578 (14)	0.0531 (7)	
O4	−0.06060 (11)	1.0138 (3)	0.27914 (11)	0.0369 (5)	
O5	−0.05492 (12)	1.3347 (3)	0.28650 (12)	0.0387 (5)	
O6	−0.19623 (11)	1.0256 (3)	0.33051 (12)	0.0427 (6)	
O1W	−0.10329 (14)	0.6837 (4)	0.38884 (13)	0.0494 (6)	
H2W	−0.1418 (13)	0.750 (5)	0.3754 (18)	0.074*	
H1W	−0.0840 (18)	0.731 (5)	0.4291 (12)	0.074*	
O2W	−0.12281 (14)	0.6700 (4)	0.18973 (12)	0.0480 (6)	
H3W	−0.123 (2)	0.766 (3)	0.1629 (15)	0.072*	
H4W	−0.122 (2)	0.577 (3)	0.1615 (14)	0.072*	
O3W	0.0390 (2)	0.1918 (4)	0.47185 (15)	0.0702 (8)	
H5W	0.047 (3)	0.286 (3)	0.444 (2)	0.105*	
H6W	0.049 (3)	0.084 (3)	0.453 (2)	0.105*	

### Atomic displacement parameters (Å^2^)

**Table:**

	*U*^11^	*U*^22^	*U*^33^	*U*^12^	*U*^13^	*U*^23^
C1	0.0464 (17)	0.033 (2)	0.0330 (15)	−0.0011 (16)	0.0041 (13)	0.0020 (17)
C2	0.0487 (18)	0.035 (2)	0.0459 (17)	0.0030 (16)	−0.0045 (14)	−0.0007 (18)
C3	0.0376 (16)	0.045 (2)	0.0480 (18)	0.0036 (16)	−0.0011 (14)	−0.0061 (18)
C4	0.051 (2)	0.038 (2)	0.075 (2)	0.0067 (18)	−0.0046 (18)	−0.008 (2)
C5	0.047 (2)	0.053 (3)	0.103 (3)	−0.003 (2)	−0.005 (2)	−0.016 (3)
C6	0.0421 (19)	0.069 (3)	0.068 (2)	0.007 (2)	−0.0073 (17)	−0.020 (3)
C7	0.056 (2)	0.070 (3)	0.055 (2)	0.013 (2)	−0.0043 (18)	0.003 (2)
C8	0.0472 (19)	0.059 (3)	0.056 (2)	−0.0014 (19)	−0.0016 (16)	0.005 (2)
C9	0.0363 (15)	0.039 (2)	0.0495 (18)	0.0025 (15)	0.0130 (14)	−0.0018 (18)
C10	0.0451 (18)	0.045 (2)	0.067 (2)	0.0010 (18)	0.0072 (17)	0.009 (2)
C11	0.051 (2)	0.067 (3)	0.070 (3)	0.010 (2)	0.0087 (19)	0.017 (2)
C12	0.044 (2)	0.083 (3)	0.067 (2)	0.002 (2)	0.0049 (18)	0.000 (3)
C13	0.050 (2)	0.075 (3)	0.066 (2)	−0.015 (2)	0.0083 (18)	−0.014 (3)
C14	0.0439 (18)	0.045 (2)	0.058 (2)	−0.0077 (17)	0.0157 (16)	−0.007 (2)
C15	0.0465 (17)	0.0297 (19)	0.0453 (17)	−0.0031 (15)	0.0128 (14)	−0.0073 (17)
C16	0.0321 (13)	0.0212 (17)	0.0317 (14)	−0.0017 (13)	0.0012 (11)	−0.0023 (15)
Ca1	0.0361 (3)	0.0204 (3)	0.0360 (3)	−0.0013 (3)	0.0055 (2)	−0.0010 (3)
Cl1	0.0981 (9)	0.0400 (7)	0.1650 (15)	−0.0089 (7)	−0.0439 (10)	0.0245 (8)
Cl2	0.0585 (6)	0.1143 (12)	0.1209 (11)	0.0061 (7)	−0.0348 (6)	−0.0308 (10)
Cl3	0.0697 (7)	0.1599 (15)	0.0775 (8)	0.0059 (8)	−0.0140 (6)	0.0030 (9)
Cl4	0.0734 (6)	0.0479 (7)	0.0945 (8)	−0.0211 (5)	0.0145 (6)	0.0040 (6)
O1	0.0637 (14)	0.0287 (14)	0.0493 (13)	−0.0065 (12)	−0.0102 (11)	−0.0002 (12)
O2	0.0485 (12)	0.0296 (14)	0.0523 (13)	−0.0008 (11)	−0.0060 (10)	0.0041 (12)
O3	0.0491 (13)	0.0330 (14)	0.0660 (15)	0.0001 (11)	−0.0159 (11)	0.0012 (13)
O4	0.0465 (11)	0.0192 (12)	0.0479 (12)	0.0012 (10)	0.0163 (10)	−0.0007 (11)
O5	0.0455 (11)	0.0198 (12)	0.0528 (13)	−0.0056 (10)	0.0143 (10)	−0.0036 (11)
O6	0.0399 (11)	0.0360 (14)	0.0535 (13)	−0.0074 (11)	0.0121 (10)	0.0018 (12)
O1W	0.0600 (15)	0.0416 (16)	0.0490 (13)	0.0065 (13)	0.0161 (11)	0.0002 (13)
O2W	0.0533 (13)	0.0359 (15)	0.0495 (13)	0.0035 (13)	−0.0031 (11)	−0.0039 (12)
O3W	0.109 (2)	0.0463 (18)	0.0554 (16)	−0.0093 (19)	0.0159 (15)	−0.0003 (15)

### Geometric parameters (Å, °)

**Table:**

C1—O1	1.248 (4)	C14—Cl4	1.732 (4)
C1—O2	1.250 (4)	C15—O6	1.424 (4)
C1—C2	1.512 (4)	C15—C16	1.520 (4)
C2—O3	1.417 (4)	C15—H15A	0.9700
C2—H2A	0.9700	C15—H15B	0.9700
C2—H2B	0.9700	C16—O5	1.246 (3)
C3—O3	1.367 (4)	C16—O4	1.251 (3)
C3—C8	1.382 (5)	C16—Ca1^i^	2.888 (3)
C3—C4	1.387 (5)	Ca1—O5^ii^	2.379 (2)
C4—C5	1.370 (5)	Ca1—O1W	2.397 (2)
C4—Cl1	1.732 (4)	Ca1—O2W	2.398 (2)
C5—C6	1.370 (6)	Ca1—O4	2.405 (2)
C5—H5	0.9300	Ca1—O1	2.485 (2)
C6—C7	1.373 (6)	Ca1—O4^iii^	2.527 (2)
C6—Cl2	1.745 (4)	Ca1—O2	2.536 (2)
C7—C8	1.367 (5)	Ca1—O5^iii^	2.550 (2)
C7—H7	0.9300	Ca1—C16^iii^	2.888 (3)
C8—H8	0.9300	Ca1—Ca1^iii^	4.0148 (6)
C9—O6	1.372 (4)	Ca1—Ca1^i^	4.0148 (6)
C9—C10	1.379 (5)	O4—Ca1^i^	2.527 (2)
C9—C14	1.392 (5)	O5—Ca1^iv^	2.379 (2)
C10—C11	1.386 (5)	O5—Ca1^i^	2.550 (2)
C10—H10	0.9300	O1W—H2W	0.818 (10)
C11—C12	1.364 (6)	O1W—H1W	0.823 (10)
C11—H11	0.9300	O2W—H3W	0.822 (10)
C12—C13	1.379 (6)	O2W—H4W	0.823 (10)
C12—Cl3	1.739 (4)	O3W—H5W	0.850 (10)
C13—C14	1.379 (5)	O3W—H6W	0.848 (10)
C13—H13	0.9300		
			
O1—C1—O2	123.5 (3)	O2W—Ca1—O1	152.75 (8)
O1—C1—C2	115.6 (3)	O4—Ca1—O1	131.10 (8)
O2—C1—C2	120.9 (3)	O5^ii^—Ca1—O4^iii^	71.29 (7)
O3—C2—C1	110.2 (3)	O1W—Ca1—O4^iii^	155.21 (8)
O3—C2—H2A	109.6	O2W—Ca1—O4^iii^	86.63 (8)
C1—C2—H2A	109.6	O4—Ca1—O4^iii^	120.50 (6)
O3—C2—H2B	109.6	O1—Ca1—O4^iii^	76.53 (8)
C1—C2—H2B	109.6	O5^ii^—Ca1—O2	128.21 (8)
H2A—C2—H2B	108.1	O1W—Ca1—O2	88.91 (8)
O3—C3—C8	126.0 (3)	O2W—Ca1—O2	153.37 (8)
O3—C3—C4	115.7 (3)	O4—Ca1—O2	79.51 (7)
C8—C3—C4	118.3 (3)	O1—Ca1—O2	51.97 (7)
C5—C4—C3	121.4 (4)	O4^iii^—Ca1—O2	97.10 (7)
C5—C4—Cl1	119.9 (3)	O5^ii^—Ca1—O5^iii^	120.33 (6)
C3—C4—Cl1	118.7 (3)	O1W—Ca1—O5^iii^	153.23 (8)
C4—C5—C6	118.9 (4)	O2W—Ca1—O5^iii^	83.90 (8)
C4—C5—H5	120.6	O4—Ca1—O5^iii^	70.50 (7)
C6—C5—H5	120.6	O1—Ca1—O5^iii^	101.18 (8)
C5—C6—C7	120.9 (3)	O4^iii^—Ca1—O5^iii^	51.11 (7)
C5—C6—Cl2	119.0 (4)	O2—Ca1—O5^iii^	78.24 (7)
C7—C6—Cl2	120.1 (3)	O5^ii^—Ca1—C16^iii^	96.11 (8)
C8—C7—C6	119.8 (4)	O1W—Ca1—C16^iii^	175.59 (8)
C8—C7—H7	120.1	O2W—Ca1—C16^iii^	85.51 (8)
C6—C7—H7	120.1	O4—Ca1—C16^iii^	95.63 (8)
C7—C8—C3	120.7 (4)	O1—Ca1—C16^iii^	88.12 (8)
C7—C8—H8	119.7	O4^iii^—Ca1—C16^iii^	25.61 (7)
C3—C8—H8	119.7	O2—Ca1—C16^iii^	86.73 (8)
O6—C9—C10	125.0 (3)	O5^iii^—Ca1—C16^iii^	25.52 (7)
O6—C9—C14	116.4 (3)	O5^ii^—Ca1—Ca1^iii^	36.91 (5)
C10—C9—C14	118.6 (3)	O1W—Ca1—Ca1^iii^	122.70 (6)
C9—C10—C11	120.6 (4)	O2W—Ca1—Ca1^iii^	78.57 (6)
C9—C10—H10	119.7	O4—Ca1—Ca1^iii^	145.47 (6)
C11—C10—H10	119.7	O1—Ca1—Ca1^iii^	75.42 (6)
C12—C11—C10	119.6 (4)	O4^iii^—Ca1—Ca1^iii^	34.50 (5)
C12—C11—H11	120.2	O2—Ca1—Ca1^iii^	118.48 (6)
C10—C11—H11	120.2	O5^iii^—Ca1—Ca1^iii^	84.01 (5)
C11—C12—C13	121.1 (4)	C16^iii^—Ca1—Ca1^iii^	59.25 (7)
C11—C12—Cl3	120.2 (4)	O5^ii^—Ca1—Ca1^i^	148.24 (6)
C13—C12—Cl3	118.7 (3)	O1W—Ca1—Ca1^i^	119.82 (6)
C14—C13—C12	119.0 (4)	O2W—Ca1—Ca1^i^	79.94 (6)
C14—C13—H13	120.5	O4—Ca1—Ca1^i^	36.54 (5)
C12—C13—H13	120.5	O1—Ca1—Ca1^i^	118.95 (6)
C13—C14—C9	121.0 (4)	O4^iii^—Ca1—Ca1^i^	84.92 (5)
C13—C14—Cl4	119.4 (3)	O2—Ca1—Ca1^i^	74.18 (5)
C9—C14—Cl4	119.6 (3)	O5^iii^—Ca1—Ca1^i^	34.07 (5)
O6—C15—C16	111.8 (2)	C16^iii^—Ca1—Ca1^i^	59.49 (7)
O6—C15—H15A	109.3	Ca1^iii^—Ca1—Ca1^i^	115.95 (3)
C16—C15—H15A	109.3	C1—O1—Ca1	93.37 (19)
O6—C15—H15B	109.3	C1—O2—Ca1	90.98 (19)
C16—C15—H15B	109.3	C3—O3—C2	117.1 (3)
H15A—C15—H15B	107.9	C16—O4—Ca1	150.93 (19)
O5—C16—O4	122.7 (3)	C16—O4—Ca1^i^	93.54 (17)
O5—C16—C15	118.6 (3)	Ca1—O4—Ca1^i^	108.96 (8)
O4—C16—C15	118.7 (3)	C16—O5—Ca1^iv^	157.43 (19)
O5—C16—Ca1^i^	61.88 (15)	C16—O5—Ca1^i^	92.60 (17)
O4—C16—Ca1^i^	60.85 (14)	Ca1^iv^—O5—Ca1^i^	109.02 (8)
C15—C16—Ca1^i^	177.18 (19)	C9—O6—C15	116.8 (3)
O5^ii^—Ca1—O1W	86.07 (8)	Ca1—O1W—H2W	112 (3)
O5^ii^—Ca1—O2W	78.00 (8)	Ca1—O1W—H1W	117 (3)
O1W—Ca1—O2W	98.71 (9)	H2W—O1W—H1W	103.9 (16)
O5^ii^—Ca1—O4	150.41 (8)	Ca1—O2W—H3W	114 (3)
O1W—Ca1—O4	84.21 (8)	Ca1—O2W—H4W	116 (3)
O2W—Ca1—O4	75.96 (8)	H3W—O2W—H4W	103.2 (16)
O5^ii^—Ca1—O1	76.38 (8)	H5W—O3W—H6W	109.8 (17)
O1W—Ca1—O1	88.67 (8)		

Symmetry codes: (i) −*x*, *y*+1/2, −*z*+1/2; (ii) *x*, *y*−1, *z*; (iii) −*x*, *y*−1/2, −*z*+1/2; (iv) *x*, *y*+1, *z*.

### Hydrogen-bond geometry (Å, °)

**Table:**

*D*—H···*A*	*D*—H	H···*A*	*D*···*A*	*D*—H···*A*
O3W—H6W···O2^ii^	0.848 (10)	2.176 (14)	3.013 (4)	169 (3)
O2W—H4W···O2^iii^	0.823 (10)	2.079 (16)	2.866 (3)	160 (4)
O2W—H3W···O1^i^	0.822 (10)	2.205 (18)	2.986 (4)	159 (4)
O1W—H1W···O3W^v^	0.823 (10)	1.927 (11)	2.745 (4)	173 (4)
O3W—H5W···O1	0.850 (10)	1.986 (12)	2.830 (4)	172 (5)
O1W—H2W···Cl4	0.818 (10)	2.88 (2)	3.530 (3)	138 (3)
O1W—H2W···O6	0.818 (10)	2.20 (2)	2.938 (3)	150 (4)

Symmetry codes: (ii) *x*, *y*−1, *z*; (iii) −*x*, *y*−1/2, −*z*+1/2; (i) −*x*, *y*+1/2, −*z*+1/2; (v) −*x*, −*y*+1, −*z*+1.

## References

[bb2] Bruker (2004). *APEX2* and *SAINT* Bruker AXS Inc, Madison, Wisconsin, USA.

[bb3] Hao, X.-M., Gu, C.-S., Song, W.-D., Ma, D.-Y. & Liu, Z.-Y. (2006). *Acta Cryst.* E**62**, m2618–m2620.

[bb4] Sheldrick, G. M. (1996). *SADABS* University of Göttingen, Germany.

[bb5] Sheldrick, G. M. (2008). *Acta Cryst.* A**64**, 112–122. 10.1107/S0108767307043930 18156677

[bb6] Song, W.-D. & Xi, D.-L. (2006). *Acta Cryst.* E**62**, m2594–m2596.

